# How to address migrant health inequity in Europe

**DOI:** 10.1016/j.lanepe.2024.100939

**Published:** 2024-05-28

**Authors:** The Lancet Regional Health – Europe

In one of the world's wealthiest and most progressive regions, migrant health remains desperately neglected. Despite 36% of all global migrant populations residing in Europe, less than half of the member states of the WHO European Region report health data for migrants, exposing a systemic failure to prioritise their wellbeing. Migrant populations face legal, structural, linguistic, and cultural barriers that systematically exclude the majority of individuals from national health-care systems with undocumented migrants bearing the brunt of discrimination. Widespread biases and inadequate cultural competency training among health-care providers treating migrants worsen disparities in access to preventive and clinical care.

This isn't just a health crisis; it is a moral reckoning. As Europe confronts this challenge, we must ask: can we reconcile migration with the principles of equity and compassion that underpin our European values? In partnership with The Lancet Migration European Regional Hub, *The Lancet Regional Health* – *Europe* presents the ‘Addressing migration and health inequity in Europe’ Series, which advocates for action to be taken in seven key areas to transition from migrant health inequity to equity and realise the 2030 Sustainable Development Goal (SDG) of leaving no one behind.

The first crucial step toward achieving equity in migrant health is to revolutionise policy formulation by embedding migrant health into national agendas and ensuring countries uphold their commitments. First, we advocate for a priority-setting approach that ensures fair allocation of health resources, targeting the most pressing migrant health needs while aligning policies with global frameworks such as the SDGs and universal health coverage (UHC). Through this approach, migrant health can be elevated from an afterthought to a central focus of national and global health agendas.

Second, extending legal entitlements to UHC for all, regardless of immigration status, ethnicity, or nationality, isn't merely a health issue—it is a moral imperative, a matter of upholding human rights and fostering socio-economic development for all. We vehemently advocate for UHC for migrant populations and in particular for undocumented migrants across the WHO European region. Undocumented migrants are unjustly denied access to even essential health-care services. Their exclusion not only jeopardises their wellbeing but also exposes the inherent non-inclusivity of our national health-care systems in Europe.

The inclusion of Ukrainian nationals under the EU Temporary Protection Directive on March 4, 2022, highlights the potential for inclusive health policies during crises. However, excluding non-Ukrainian nationals fleeing Ukraine under this act reveals European Union double standards, contradicts universal human rights principles, and emphasizes the urgent need for governments to be held accountable for upholding the rights of all migrants, regardless of origin or circumstances.

Third, the current vaccination approach in European migrant populations falls short, perpetuating health inequities and undermining UHC. Migrants often miss routine vaccination programmes on arrival, leaving them susceptible to infectious disease outbreaks. This not only jeopardizes migrant health but also public health overall. To tackle this issue, we advocate for strengthening life-course immunisation programmes for migrants. This includes integrating catch-up vaccinations and aligning migrant populations with European vaccination schedules on or shortly after arrival.

Fourth, at the forefront of addressing health equity in Europe is migrant-sensitive health care, which emphasizes accessibility, acceptability, quality, and trust. European health systems continue to fall short of providing care that meets the diverse needs of migrants with the same standard as the host population. Innovative approaches, with involvement of social and cultural sciences, are imperative for adapting clinical care and health services to the increasing diversity of European societies.

Fifth, the inclusion of people with lived experience in health research is increasing. However, refugees and migrants are frequently excluded from studies concerning their own health. This exclusion perpetuates a top-down approach, leading to biased outcomes and exacerbating disparities. We advocate for a paradigm shift in refugee and migrant health research to make participatory health research a routine practice to ensure their voices are heard and research is guided by migrants and their aspirations.

Sixth, unaccompanied refugee children, asylum seekers, and undocumented migrants often face heightened health needs due to migration experiences. Yet, racism, xenophobia, and discriminatory policies in European health systems exacerbate these issues, violating international human rights obligations that hinder integration and harm mental health, especially for unaccompanied refugee children. We urge prioritisation of research on the impact of health system-produced racism, xenophobia, and discrimination on child migrants and advocate for robust data collection to inform evidence-based, equitable health policies.

Seventh, capacity building in migration and health within higher education is crucial for tackling the complex health inequities faced by migrant populations. By equipping future health-care professionals with relevant knowledge and skills, they will be empowered to effectively meet migrants' unique needs, considering cultural nuances. Comprehensive capacity building efforts should involve both top-down leadership initiatives and active bottom-up engagement of migrants, teachers, and students.

As right-wing populism and xenophobia surge in Europe, integrating migrant health into all policies is not just necessary—it's a moral mandate to safeguard the wellbeing of all populations. With most European countries signatories to the European Convention on Human Rights, acknowledging and addressing migrants' health needs, irrespective of origin or status, is a duty. Europe must go beyond legal obligations and embrace its moral responsibility to ensure equitable access to health-care for all.

